# Preterm toddlers have low nighttime sleep quality and high daytime activity

**DOI:** 10.1038/s41598-022-24338-8

**Published:** 2022-11-21

**Authors:** Yoko Asaka, Yusuke Mitani, Hidenobu Ohta, Takayo Nakazawa, Rika Fukutomi, Kyoko Kobayashi, Mayuko Kumagai, Hitomi Shinohara, Michiko Yoshida, Akiko Ando, Yuko Yoshimura, Machiko Nakagawa, Yoshihisa Oishi, Masato Mizushima, Hiroyuki Adachi, Yosuke Kaneshi, Keita Morioka, Yoshitaka Seto, Rinshu Shimabukuro, Michio Hirata, Takashi Ikeda, Miwa Ozawa, Masahiro Takeshima, Atsushi Manabe, Tsutomu Takahashi, Kazuo Mishima, Mitsuru Kikuchi, Hitoshi Yoda, Isao Kusakawa, Kazutoshi Cho

**Affiliations:** 1 Department of Maternal and Child Health Nursing, Mie University Graduate School of Medicine, Edobashi 2-174, Tsu, 514-8507 Japan; 2 Department of Pediatrics, Kanazawa University, 13-1 Takara-Machi, Kanazawa, 920-8640 Japan; 3 Department of Neuropsychiatry, Akita University Graduate School of Medicine, 1-1-1 Hondo, Akita, 010-8543 Japan; 4 Department of Sleep-Wake Disorders, National Institute of Mental Health, National Center of Neurology and Psychiatry, 4-1-1 Ogawa-Higashi-Cho, Kodaira, Tokyo 187-8553 Japan; 5 Department of Psychiatry, Asai Hospital, 38-1 Togane, Chiba, 283-0062 Japan; 6 Department of Occupational Therapy, Akita University Graduate School of Medicine, 1-1-1 Hondo, Akita, 010-8543 Japan; 7 Maternity and Perinatal Care Center, Hokkaido University Hospital, N15, W7, Kita-Ku, Sapporo, 060-8638 Japan; 8 Department of Pediatric Nursing, Graduate School of Nursing Science, St. Luke’s International University, 10-1 Akashi-Cho, Chuo-Ku, Tokyo, 104-0044 Japan; 9 Department of Nursing, Akita University Graduate School of Medicine, 1-1-1 Hondo, Akita, 010-8543 Japan; 10 Graduate School of Nursing, Hyogo University, 2301 Shinzaike, Hiraoka-Cho, Kakogawa, 675-0195 Japan; 11 Department of Nursing, Faculty of Health Sciences, Hokkaido University, N12, W5, Kita-Ku, Sapporo, 060-0812 Japan; 12 Institute of Human and Social Sciences, Kanazawa University, Kakuma-Machi, Kanazawa, 921-1192 Japan; 13 Research Center for Child Mental Development, Kanazawa University, 13-1 Takara-Machi, Kanazawa, 920-8640 Japan; 14 Department of Pediatrics, St. Luke’s International Hospital, 9-1 Akashi-Cho, Chuo-Ku, Tokyo, 104-8560 Japan; 15 Department of Neonatology, Toho University Omori Medical Center, 6-11-1 Omori-Nishi, Ota-Ku, Tokyo, 143-8541 Japan; 16 Department of Pediatrics, Japanese Red Cross Medical Center, 4-1-22 Hiroo, Shibuya-Ku, Tokyo, 150-8935 Japan; 17 Department of Neonatology, Sapporo City Hospital, N11, W13, Chuo-Ku, Sapporo, 060-8604 Japan; 18 Department of Pediatrics, Akita University Graduate School of Medicine, Hondo 1-1-1, Akita, 010-8543 Japan; 19 Department of General Pediatrics and Interdisciplinary Medicine, National Center for Child Health and Development, 2-10-1 Ohkura, Setagaya-Ku, Tokyo, 157-8535 Japan; 20 Department of Child Studies, Faculty of Human Sciences and Design, Japan Women’s University, 2-8-1 Mejirodai, Bunkyo-Ku, Tokyo, 112-8681 Japan; 21 Department of Pediatrics, Hokkaido University Graduate School of Medicine, N15, W7, Kita-Ku, Sapporo, 060-8638 Japan; 22 Department of Psychiatry and Neurobiology, Kanazawa University, 13-1 Takara-Machi, Kanazawa, 920-8640 Japan

**Keywords:** Medical research, Paediatric research

## Abstract

A number of studies have been made on the sleep characteristics of children born preterm in an attempt to develop methods to address the sleep problems commonly observed among such children. However, the reported sleep characteristics from these studies vary depending on the observation methods used, i.e., actigraphy, polysomnography and questionnaire. In the current study, to obtain reliable data on the sleep characteristics of preterm-born children, we investigated the difference in sleep properties between 97 preterm and 97 term toddlers of approximately 1.5 years of age using actigraphy. Actigraphy units were attached to the toddlers’ waists with an adjustable elastic belt for 7 consecutive days, and a child sleep diary was completed by their parents. In the study, we found that preterm toddlers had more nocturnal awakenings and more daytime activity, suggesting that preterm-born children may have a different process of sleep development in their early development.

Preterm infants experience the dramatic change from intrauterine to extrauterine environment with more physiological immaturity than term infants, which is considered to lead to inappropriate developmental processes of the brain and other organs at early developmental stages^1^. Long-term hospitalization in an artificial environment, such as a neonatal intensive care unit (NICU), has also been reported to give additional negative impacts on the sleep development of preterm infants^2,3^, possibly causing behavioral problems during early childhood^4^ or leading to parent depression during child raising^5^. To investigate the effects of preterms’ physiological prematurity and NICU environments on preterm infants’ sleep development, several studies have examined the difference in sleep characteristics between preterm and term infants^4,6–13^. Among them a polysomnographic study has reported no difference in the sleep structure between term and preterm infants at the gestational ages of 38 to 55 weeks^9^. In addition, previous sleep surveys using sleep diaries and questionnaires completed by caregivers have also reported no difference in the sleep development between preterm and term toddlers aged one year and over^6,7,10^. However, other sleep studies have reported a difference in the sleep development between preterm and term toddlers^4,8,11–13^.

Infant circadian sleep patterns of day and nighttime sleep incorporating ultradian rhythms of short periods of sleep start to be established by approximately three months of age^14^. In addition, the biphasic sleep pattern of nap and nighttime sleep among young toddlers is established at the latest by 1.5 years of age, while nap duration gradually decreases until napping ceases by 5 years of age^15–20^. In the current study, we investigate the difference in sleep characteristics between preterm and term toddlers in order to confirm whether preterm toddlers have a different sleep development process from that of term toddlers at 1.5 years of age, when their fundamental sleep structure normally takes effect.

## Results

### Comparison of birth profiles and clinical features between preterm and term toddlers

The comparison of birth profiles and clinical features between preterm and term toddlers of approximately 1.5 years of age is shown in Table 1. Ninety-seven preterm and ninety-seven term toddlers were enrolled in the present study. Significant differences were detected in all parameters except for gender, birth order and corrected months of age at actigraph recording. At both birth and time of study, the preterm group had significantly lower weights, shorter heights, and smaller head circumferences than the term group. Z-scores also indicate that the preterm toddlers had reduced heights, weights and head circumferences in comparison with the term toddlers at both birth and 1.5 years corrected age (Table 1).

**Table 1:** Demographic characteristics of participants (number (%) or mean ± SD).

	Preterm (n = 97)	Term (n = 97)	p-value
**Gestational age at birth (weeks)**	28.8 ± 2.5	39.6 ± 1.1	0.001
**Gender**			
Boys	43 (44.3%)	45 (46.4%)	0.773
Girls	54 (55.7%)	52 (53.6%)	
**Type of delivery**			
Caesarean section	90 (92.8%)	21 (21.6%)	0.001
Vaginal delivery	7 (7.2%)	76 (78.4%)	
**Birth order**			
First born	65 (67.0%)	68 (70.1%)	0.643
Subsequent born	32 (33.0%)	29 (29.9%)	
SGA	38 (39.2%)	2 (2.1%)	0.001
**Apgar score**			
1-min	5.9 ± 2.3	8.1 ± 1.1	0.001
5-min	8.0 ± 1.6	9.1 ± 0.6	0.001
**Birth weight (g)**	1010.9 ± 292.0	3089.4 ± 379.6	0.001
**Birth height (cm)**	35.4 ± 3.8	49.7 ± 1.7	0.001
**Birth head circumference (cm)**	25.5 ± 2.4	33.6 ± 1.9	0.001
**Z score for birth weight (g)**	−1.24 ± 1.2	0.18 ± 0.9	0.001
**Z score for birth height (cm)**	−0.93 ± 1.2	0.33 ± 0.8	0.001
**Z score for head circumference (cm)**	−0.43 ± 0.8	0.10 ± 0.9	0.001
**Corrected months of age at actigraph recording**	19.5 ± 0.9	19.2 ± 0.8	0.070
**18-month weight (kg)**	9.4 ± 1.0	10.4 ± 1.0	0.001
**18-month height (cm)**	78.2 ± 2.8	80.3 ± 2.7	0.001
**18-month head circumference (cm)**	46.4 ± 1.7	47.3 ± 1.2	0.001
**Z score for 18-month weight (kg)**	−0.89 ± 0.9	0.06 ± 0.9	0.001
**Z score for 18-month height (cm)**	−0.91 ± 1.0	−0.11 ± 1.0	0.000
**Z score for 18-month head circumference (cm)**	−0.15 ± 1.1	0.45 ± 0.8	0.001

SGA: Small for gestational age.

### Comparison of sleep parameters and daytime activity between preterm and term toddlers

**Figure 1:**
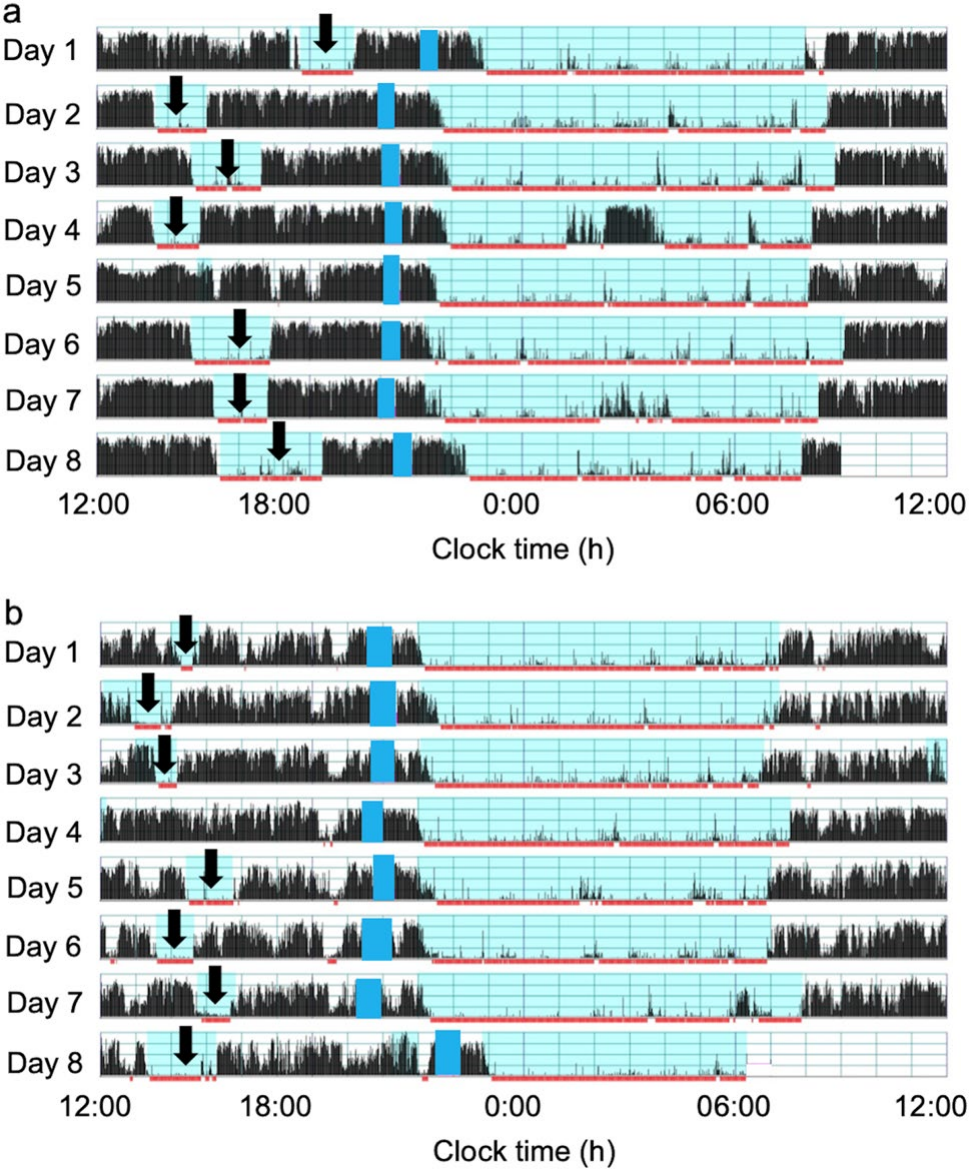
The representative actograms of both preterm (**a**) and term (**b**) toddlers of approximately 1.5 years of age. Figures (**a**) and (**b**) show the daytime activity and nighttime sleep patterns of two individuals chosen as typical examples to represent their corresponding groups. The vertical axis shows the 7 consecutive observation days and the horizontal axis shows the course of each 24 h day from 00:00 h (12:00 am) to 24:00 h (12:00 am). Activity counts per minute are represented by the height of the vertical black bars on each actogram. The arrows and the blue rectangles indicate naps and bathing periods, respectively. The red underlines are the periods that were automatically judged as sleep periods by the actigraph software. Note that, compared to the term toddlers (**b**), the preterm toddlers (**a**) had more nocturnal awakenings and daytime activity despite having almost no difference in sleep onset and wake time.

The comparison of sleep parameters and activity data between the two groups at approximately 1.5 years of age is shown in Tables 2, 3 and Fig. 1. The three sleep parameters of nocturnal awakenings, WASO (wake after sleep onset) and sleep efficiency were significantly different between the two groups. The preterm toddlers had more nocturnal awakenings (*p* = 0.001), more WASO (*p* = 0.002) and lower sleep efficiency (*p* = 0.004) than the term toddlers (Mann–Whitney U test). The preterm toddlers also had more daytime activity than the term toddlers (*p* = 0.037, Mann–Whitney U test). Regarding nonparametric sleep variables, M10 was significantly higher in the preterm toddlers than the term toddlers (*p* = 0.050, i.e., *p* = 0.0498 < 0.05, Mann–Whitney U test), which suggests that the preterm toddlers were more active than the term toddlers during wake periods while no significant differences were detected in the other nonparametric sleep parameters.

**Table 2:** Comparison of sleep parameters and daytime activity between preterm and term toddlers (median (IQR)).

Sleep/activity parameters	Preterm (n = 97)	Term (n = 97)	p-value
Sleep onset time	21:29 (20:57,21:56)	21:32 (21:03, 22:01)	0.419
Wake time	7:01 (6:27, 7:25)	7:08 (6:26, 7:32)	0.361
Sleep latency (min)	27.0 (19.2, 35.9)	26.0 (19.5, 34.4)	0.678
Nocturnal awakenings	14.3 (10.0, 17.0)	11.0 (8.0, 14.6)	0.001
WASO (min)	71.9 (48.6, 100.4)	48.3 (29.2, 88.5)	0.002
Sleep efficiency (%)	88.0 (82.6, 91.5)	91.0 (83.8, 94.7)	0.004
Total sleep duration (h)	10.3 (9.4, 10.7)	10.4 (9.8, 10.9)	0.112
Nighttime sleep duration (h)	8.3 (7.5, 8.7)	8.4 (7.7, 9.0)	0.075
Nap duration (h)	2.0 (1.7, 2.3)	1.9 (1.6, 2.3)	0.546
Nap onset time	12:27 (11:58, 12:59)	12:42 (12:00, 13:31)	0.245
Nap end time	14:54 (14:24, 15:29)	14:54 (14:26, 15:34)	0.585
Daytime activity (counts/min)	239.2 (227.1, 252.5)	234.7 (224.6, 244.2)	0.037
Nighttime activity (counts/min)	25.1 (19.1, 30.1)	22.6 (18.9, 30.0)	0.168

WASO: wake after sleep onset.

**Table 3:** Comparison of nonparametric sleep parameters between preterm and term toddlers (median (IQR)).

Nonparametric sleep parameters	Preterm (n = 97)	Term (n = 97)	p-value
IS	0.57 (0.49, 0.71)	0.63 (0.48, 0.73)	0.700
IV	0.54 (0.50, 0.59)	0.54 (0.50, 0.60)	0.668
L5 counts	18.7 (13.4, 25.1)	17.9 (11.9, 22.9)	0.264
M10 counts	195.7 (184.3, 213.7)	192.1 (176.3, 205.4)	0.050
RA	0.82 (0.78, 0.87)	0.83 (0.79, 0.87)	0.381
L5 onset	21:38 (21:02, 22:33)	21:51 (20:50, 22:53)	0.432
M10 onset	7:03 (6:16, 7:58)	7:08 (3:58, 11:06)	0.999

IS: Interdaily stability, IV: Intradaily variability, L5 counts: activity counts of the least active 5 h of the day, M10 counts: activity counts of the most active 10 h of the day, RA: (M10 counts – L5 counts) / (M10 counts + L5 counts), L5 onset: activity onset time of the least active 5 h of the day, M10 onset: activity onset time of the most active 10 h of the day.

In addition, ANCOVA analysis revealed that the bathing-related factors (the covariates) of bathing duration, bath-start time, and bath-end time did not affect the statistical difference in nocturnal awakenings, WASO, sleep efficiency, or daytime activity between preterm and term toddlers (*p* > 0.05). The preterm toddlers were additionally separated into either an SGA (small-for-gestational-age) or non-SGA group, and sleep parameters and activity data were then statistically compared between these two groups. No significant difference in sleep parameters or activity data was found between the SGA and non-SGA group (*p* > 0.05, Mann–Whitney U test).

## Discussion

The present study makes four significant findings concerning the sleep properties and daytime activity levels of preterm and term toddlers at approximately 1.5 years of age. First, sleep analysis indicates that the preterm toddlers in our study had a lower quality of nighttime sleep than the term toddlers. Compared to the term toddlers, the preterm toddlers had more nocturnal awakenings, more WASO, and lower sleep efficiency, indicating that the preterm toddlers did not maintain as well a consolidated nighttime sleep as the term toddlers (Table 2 and Fig. 1), although no difference in nighttime sleep duration was detected between the two groups. This result is partly in line with our previous actigraphic study^11^, in which preterm infants of 1 year of age had more nocturnal awakenings and shorter nighttime sleep duration than term infants. A subsequent actigraphic study^12^ of preterm toddlers of approximately 14 months of age, however, demonstrated that preterm toddlers had more nocturnal awakenings than term toddlers but found no difference in nighttime sleep duration. Using actigraphy, Gössel-Symank et al.^8^ also reported that preterm toddlers of 20 months of age had a longer total period of nighttime movement. The current actigraphic study fits well with a polysomnographic study^21^ in which children aged 5–12 years who had been born prematurely were found to have more WASO. In contrast, previous questionnaire studies reported no difference in nocturnal awakenings or nighttime sleep duration between preterm and term children of up to 10 years of age^6,7,10^. The reason for the discrepancies between these studies and ours could be because our study employed actigraphy for sleep assessment while the conflicting previous studies did not. Actigraphy is generally regarded as superior to parental reports in estimating child sleep–wake activity because some parents tend to underestimate child waking frequencies and durations^22,23^. Despite this, the results of the current study are partially inconsistent with those of the above-mentioned study by Gössel-Symank et al.^8^, which also employed actigraphy. The present study detected no difference in nighttime sleep duration between preterm and term toddlers while the study by Gössel-Symank et al.^8^ reported that preterm toddlers had shorter nighttime sleep duration. The reason for this discrepancy may come from the difference in the sample size between the two studies (97 term and 97 preterm toddlers of approximately 19 months of age in the current study; 17 term and 8 preterm toddlers of approximately 20 months of age in the study by Gössel-Symank et al.^8^).

The second significant finding was that the preterm toddlers had more daytime activity than the term toddlers, which we did not focus on in our previous study^11,12^. Compared to the term toddlers, the preterm toddlers had more activity during daytime (Table 2 and Fig. 1), indicating a possibility that preterms are more prone to hyperactivity than term toddlers. This is consistent with the results of the M10 counts in the present study that indicate the preterm toddlers were more active than the term toddlers during the most active 10 h of the day, approximately between 7:00 and 17:00 (Table 3). This is also in agreement with the previous actigraphic study by Gössel-Symank et al.^8^, which found that preterm toddlers of 20 months of age had a shorter daytime rest duration than correspondingly-aged term toddlers. A separate questionnaire study also reported that preterm children of 2 years of age experienced sleep disturbances, such as nocturnal awakenings and restlessness, which were associated with poor attention and hyperactivity^4^. These results may support a hypothesis that attention deficit/hyperactivity disorder (ADHD) is more common among people born as preterm infants^24–26^.

Third, Z-scores indicate that the preterm toddlers had reduced heights, weights and head circumferences in comparison with the term toddlers at 1.5 years corrected age (Table 1) ^27,28^, indicating that the preterm toddlers had not caught up in growth with the term toddlers at that stage. This is consistent with several previous studies in which 19–40% of preterm infants of birth weight < 1,500 g still had a weight, length, and head circumference less than the 10th percentile at 18–24 months of corrected age^29,30^. Since only infants with healthy status were included in the present study, we assume that the differences in anthropometric measurements do not have any obvious clinical significance at this age.

Fourth, none of the sleep parameters or activity data between the SGA or non-SGA group showed any significant difference (*p* > 0.05, Mann–Whitney U test), indicating that there is no correlation between the sleep parameters of 1.5-year-corrected-age preterm toddlers and SGA status at birth. Therefore, intrauterine growth restriction (IUGR) may not have affected the sleep development of the young toddlers in this study. No study on comparison of sleep development between SGA and non-SGA preterm toddlers has been previously reported. However, an actigraphic study comparing children born as term infants with IUGR and those without IUGR has revealed that term children with IUGR aged 4–7 years have shorter nighttime sleep duration than those without IUGR^31^.

There are six matters concerning the current study that warrant consideration. First, although the sleep habits of toddlers are affected by those of their parents, especially mothers^32^, the present study did not investigate the sleep habits of the parents themselves. Secondly, the sleep habits of toddlers are also affected by socio-cultural environments, socioeconomics, individual family ethics, income and the educational backgrounds of parents^33^. The details of these were not obtained in the present study due to privacy concerns. Third, polysomnography, the gold standard for sleep evaluation, was not employed for the current study. A previous study, however, has reported that actigraphy, which was used in the present study, had a sensitivity of 87.7% and a specificity of 76.9% for children aged 1–2 years, indicating that actigraphy has adequate accuracy for detecting wake status at night^34^. Fourth, the current study did not explore possible solutions for the sleep problems observed among preterm toddlers, such as increased nocturnal awakenings, although several pre-bedtime sleep promotion routines have already been found to be effective for the improvement of toddlers’ nighttime sleep and the reduction of parents’ depression^35^. Fifth, since this is not a longitudinal study and also diagnosis of ADHD usually is not made until the age of around 8 years of age, we cannot conclude that high daytime activity of preterm toddlers contributes to future occurrence of ADHD. Sixth, since the present study did not gather information on sleep routines^36^, we are not able to analyze the effects of bedtime routines on the difference in sleep parameters between preterm and term toddlers. However, ANCOVA analysis revealed that the bathing-related factors (the covariates) of bathing duration, bath-start time, and bath-end time did not affect the statistical difference in nocturnal awakenings, WASO, sleep efficiency, or daytime activity between preterm and term toddlers (p > 0.05).

In summary, our findings indicate a possibility that preterm toddlers have lower nighttime sleep quality and more daytime activity than term toddlers. Further studies should be explored to find strategies for early intervention against such possible sleep problems in preterm toddlers.

## Methods

### Participants

Preterm and term toddlers of approximately 1.5 years of age were recruited from multiple research facilities; Hokkaido University Hospital (Sapporo, Japan), Sapporo City Hospital (Sapporo, Japan), St. Luke’s International Hospital (Tokyo, Japan), Toho University Hospital (Tokyo, Japan), Japanese Red Cross Medical Center (Tokyo, Japan), Kanazawa University Hospital (Kanazawa, Japan) and Akita University Hospital (Akita, Japan), as previously described^19,20^. Inclusion criteria were 1) the absence of chromosomal or other major genetic abnormalities, suspected neuromuscular disorders, intraventricular hemorrhage (> grade 1) or significant chronic lung disease (CLD) for both groups of toddlers, 2) birth weight of less than 1,500 g (very low birth weight) and born at less than 37 gestational weeks for the preterm infants, 3) born at between 37 and 42 gestational weeks for term toddlers. Exclusion criteria were parental language difficulties. Age correction was carried out for preterm toddlers to compare their sleep parameters and activity data with comparable term toddlers. Age correction was performed in the following way: the duration between expected birth date and actual birth date was subtracted from the actual age.

105 preterm and 129 term toddlers who met the inclusion criteria were enrolled in the study. Out of those, 4 preterm and 23 term toddlers were excluded for invalid data due to technical problems with the activity recording devices or incomplete description of sleep diary, reducing the number of preterm and term toddlers to 101 and 106 respectively. Of those eligible preterm and term toddlers, a further 4 preterm and 9 term toddlers were excluded for age matching. The final sample thus consisted of 97 preterm (45 boys, 52 girls) and 97 term (43 boys, 54 girls) toddlers.

The ethics committees of all research facilities described above (Hokkaido University Hospital, Sapporo City Hospital, St. Luke’s International Hospital, Toho University Hospital, Japanese Red Cross Medical Center, Kanazawa University Hospital and Akita University Hospital) approved the study protocol (UMIN000021153) and all procedures were carried out in accordance with the approved guidelines. Informed written consent was obtained from the toddlers’ parent/s.

### Sleep and activity assessment

For sleep assessment, we used Actigraphs (Micro-mini RC, Ambulatory Monitoring Inc., Ardsley, NY, USA) and sleep diaries, as previously described^19,20^. Actigraphs, which are wristwatch-like devices, have been widely used for sleep assessment including that of children. They consist of a piezoelectric sensor sensitive to accelerations of 0.01 g per second and above and record each detected activity count. Actigraphs were set for 1-min epochs and set in zero-crossing mode.

Mothers were instructed to leave this device attached to their toddler’s waist with an adjustable soft cloth belt for 7 days, taking it off only for bathing. Study period and actigraph body-attachment location for this study were decided based on the findings of previous studies^37–39^. The activity data retrieved from the Actigraphs were downloaded to a personal computer using the automatic Actigraph interface unit and ActMe software (ver. 3.10.0.3, Ambulatory Monitoring Inc., Ardsley, NY, USA). Activity data were then analyzed using Action-W software (ver. 2.4.20, Ambulatory Monitoring Inc., Ardsley, NY, USA) with the Sadeh algorithm^34^ and sleep/activity parameters (sleep onset time, wake time, sleep latency, nocturnal awakenings, WASO, sleep efficiency, total sleep duration, nighttime sleep duration, nap duration, nap onset time, nap end time, daytime activity, and nighttime activity) were calculated. Nonparametric sleep parameters (IS, IV, RA, L5, L5 onset, M10, and M10 onset) were also calculated to assess circadian sleep–wake rhythmicity using the ‘npar ACT’ package for R^40^.

The parents were asked to complete a sleep diary documenting the nap and nighttime sleep patterns of the toddlers. The sleep diary was composed of seven 24-h single-sheet schedules and included information about the toddlers’ sleep schedules and sleep quality, for example bedtimes, wake times, and nocturnal awakenings, as well as unusual external motions, such as being moved in a car or a stroller. Data from sleep diaries were used to specify bedtimes and wake times, and also nap start and end times. These data were used to confirm the accuracy of the actigraphy data and also to confirm that the actigraph had not been accidentally removed from the infants during sleep or activity. Parents were also asked about the demographic data of their children. Clinical data of each participant were collected from their medical records.

### Statistical analysis

Power analysis using G*Power^41^ in a pilot study showed that, to test a group difference with an effect size of 0.5, a significance level of α = 0.05 and a power of 0.80, a sample size of 102 (51 toddlers in each group) was necessary when using a two-independent-sample t-test while a sample size of 106 (53 toddlers in each group) was necessary when using a two-independent-sample Mann–Whitney U test. Since the normality of the sleep parameters was not confirmed by the Kolmogorov–Smirnov normality test, we carried out a Mann–Whitney U test to compare the estimated sleep parameters and activity data between the preterm and term group. For statistical analysis of the demographic data of the participants, a t-test was performed for continuous variables and a chi-square test or Fisher's exact test was performed for other parameters. ANCOVA was used to examine the effects of the bathing-related factors (the covariates) of bathing duration, bath-start time, and bath-end time on the statistical difference in nocturnal awakenings, WASO, sleep efficiency, and daytime activity between preterm and term toddlers. The normality of the distribution was confirmed before performing ANCOVA analysis. It was also confirmed that n = 75 was sufficient to perform ANCOVA analysis using G*Power^39^ for the comparison of sleep variables between different bedtime routines. All statistical procedures were carried out using IBM SPSS Ver 27.0 (IBM Corp. Armonk, NY, USA), and the setting for all significant probability levels was *p* < 0.05.
